# Characterization of the Mycoremediation of *n*-Alkanes and Branched-Chain Alkanes by Filamentous Fungi from Oil-Polluted Soil Samples in Kazakhstan

**DOI:** 10.3390/microorganisms11092195

**Published:** 2023-08-30

**Authors:** Mariam Gaid, Doreen Pöpke, Anne Reinhard, Ramza Berzhanova, Togzhan Mukasheva, Tim Urich, Annett Mikolasch

**Affiliations:** 1 Institute of Microbiology, University Greifswald, Felix-Hausdorff-Straße 8, 17489 Greifswald, Germany; 2 Department of Biology and Biotechnology, Al-Farabi Kazakh National University, Al-Farabi Ave. 71, Almaty 050040, Kazakhstan

**Keywords:** alkanes, di-terminal oxidation, filamentous fungi, mycoremediation, oil degradation, pollution

## Abstract

For decades, researchers have focused on containing terrestrial oil pollution. The heterogeneity of soils, with immense microbial diversity, inspires them to transform pollutants and find cost-effective bioremediation methods. In this study, the mycoremediation potentials of five filamentous fungi isolated from polluted soils in Kazakhstan were investigated for their degradability of *n*-alkanes and branched-chain alkanes as sole carbon and energy sources. Dry weight estimation and gas chromatography–mass spectrometry (GC-MS) monitored the growth and the changes in the metabolic profile during degradation, respectively. *Penicillium javanicum* SBUG-M1741 and SBUG-M1742 oxidized medium-chain alkanes almost completely through mono- and di-terminal degradation. Pristane degradation by *P. javanicum* SBUG-M1741 was >95%, while its degradation with *Purpureocillium lilacinum* SBUG-M1751 was >90%. *P. lilacinum* SBUG-M1751 also exhibited the visible degradation potential of tetradecane and phytane, whereby in the transformation of phytane, both the mono- and di-terminal degradation pathways as well as α- and ß-oxidation steps could be described. *Scedosporium boydii* SBUG-M1749 used both mono- and di-terminal degradation pathways for *n*-alkanes, but with poor growth. Degradation of pristane by *Fusarium oxysporum* SBUG-M1747 followed the di-terminal oxidation mechanism, resulting in one dicarboxylic acid. These findings highlight the role of filamentous fungi in containing oil pollution and suggest possible degradation pathways.

## 1. Introduction

The heterogeneity of soils allows for immense microbial diversity [1]. Microbial “hotspots” are essential to this heterogeneity, although they make up less than 1% of the total soil volume [2,3]. They are characterized by many microorganisms in the soil that tend to form colonies, biofilms, or aggregates [3,4].

Oil contamination has toxic effects on plants and animals [5,6,7,8]. The global crude oil export value of Kazakhstan in 2017 was USD 26,584 million [9], making Kazakhstan the fourth most important oil supplier to Germany, with 4.3 million tons in 2017, and one of the first oil supporters in 2023 [9,10]. The production, transport, processing, and consumption of crude or mineral oil products can cause significant environmental pollution. In Germany, different strategies are being taken to combat oil spills [11].

One possible method for containing oil contamination in water is the use of oil booms. These are placed around an oil slick and prevent further spreading. However, several factors need to be considered, such as flow rate, width, and depth of the respective body of water [12]. To make oil retention even more efficient, the use of multiple booms is recommended [12]. Oil skimming ships can also be used to remove floating oil from the water surface. Additionally, oil slicks can be burned, but not every type of oil can be removed by this method and is influenced, among other things, by the type of oil and meteorological and oceanographic conditions [13]. However, burning crude oil also releases carbon dioxide into the atmosphere. By increasing carbon dioxide concentration in the atmosphere, the emission of heat into space is reduced, causing global warming [14]. Further research is needed to determine the extent to which combustion residues affect marine ecosystems. Another method for combating oil contamination is the use of chemical dispersants, which can be spread over a large area by aircraft. This disperses the oil into small droplets, making it available to oil-degrading microorganisms [15].

Bioremediation is a process during which microorganisms are used for oil degradation. However, this method has not yet been fully investigated and requires deeper research. Among the oil-degrading microorganisms, bacteria have been studied and described in particular [16,17,18,19]. Fungi are spread worldwide and dominate the topsoil with more than 80% of their biomass, with only about 70,000 of the approximately 1.5 million fungal species known so far [20]. In addition to their specific metabolic capabilities, they could be of great interest in terms of terrestrial oil degradation.

Several fungal isolates have been recently identified for their ability to use crude oil as a sole source of energy and carbon [21,22,23,24]. In 2007, Kazakhstan produced over 68 million tons of crude oil, of which over 50 million tons were exported [25]. In Kazakhstan, the oil industry mainly contaminates the soil through leaking oil from pipelines, boreholes, and/or leaky storage so the first 10 m of soil layers near older oil fields were reported to be saturated with oil [26]. Previously, microbial diversity of polluted soils in Kazakhstan and their potential for the degradation of petroleum components were investigated [27].

In the current study, the cultivation and turnover performance of five fungal isolates were examined for their degradation potential of branched-chain and un-branched n-alkanes as model compounds of crude oil components. This offers an environmental remediation tool for dealing with the serious impacts of petroleum hydrocarbons to restore ecosystems. Offering this strategy, scientists can implement filamentous fungi to reduce the drawbacks of oil pollution, which can be extended to break a wider range of complex hydrocarbons.

## 2. Materials and Methods

### 2.1. Fungal Cultures

In the present study, five different filamentous fungi were investigated for their microbial degradation potential of pollutants. All strains were isolated from soil samples collected in Kazakhstan in a previous study by Müller [27] and were taken from the strain collection of the Department of Biology at the University of Greifswald (SBUG) for the present study—strains *Penicillium javanicum* SBUG-M1741 (internal code 15/P17/K2), *Penicillium javanicum* SBUG-M1742 (internal code 17/P17/K1), *Scedosporium boydii* SBUG-M1749 (internal code 68/P38/K1), and *Fusarium oxysporum* SBUG-M1747 (internal code 11/P27/K1) were isolated from an oil-contaminated soil sample (50°20′38.2″ N, 57°05′11.0″ E) at the oil storage facility in the Aktöbe region, while strain *Purpureocillium lilacinum* SBUG-M1751 (internal code 71/P36/K1) was isolated from a soil sample (43°20′36.8″ N, 76°56′30.7″ E) collected in a park near the Almaty train station.

Before each experiment, the filamentous fungi were transferred from a well-grown malt agar (MAg) plate to a new medium by using three agar pieces (1 × 1 cm) with fungal growth. The plates were then incubated at 30 °C for 7 days to ensure sufficient biomass growth for the upcoming experiments. To avoid contamination with bacteria, chloramphenicol (0.3 mg/mL, SERVA, Heidelberg, Germany) was added to MAg. Afterward; the plates were stored at approximately 4 ± 0.5 °C.

Mineral salt medium for fungi (MSMHe) was used for cultivation, which consists of a basal medium [28] and a solution of trace elements [29]. Before autoclaving, the pH was adjusted to 5.4. A vitamin stock solution [30] was added to the MSMHe at a final concentration of 1% after sterile filtration using 0.22 µm filter (Fisherbrand, Schwerte, Germany).

MSMHe plates are used to determine the growth of fungi on different substrates. For this purpose, 17 g/L agar–agar (Invitrogen-Fisher scientific, Dreieich, Germany) was added to the MSMHe before autoclaving. After autoclaving and cooling to approximately 60 °C, the vitamin stock solution was added at a final concentration of 1% *v*/*v*. The mixed medium was then poured into sterile glass petri dishes.

MAg plates were also used for short-term strain maintenance and cell cultivation. It was prepared by dissolving 25 g organic malt (Villa Natura Gesundprodukte GmbH, Kirn, Germany) and 18 g agar–agar in 1 L distilled water using a pressure cooker, followed by adjusting the pH to 5.5 and adding chloramphenicol (0.3 mg/mL). The MAg was poured into sterile petri dishes after autoclaving and cooling to approximately 60 °C. Organic malt 2.5% *w*/*v* in water was used to prepare malting broth for the pre-cultures of the biotransformation experiments. The pH was adjusted to 5.5 before autoclaving.

### 2.2. Substrates

All substrates were purchased from Sigma-Aldrich (Taufkirchen, Germany). For the incubation experiments, three substrates were used: tetradecane, pristane, and phytane. All substrates were sterilized by filtration (Sartorius Minisart SRP 25, pore size of 0.2 µm, Sartorius Stedim Biotech, Göttingen, Germany).

### 2.3. Strain Identification

#### 2.3.1. Microscopy

All five fungal strains were examined under a microscope (LEICA DM 2500 LED, Wetzlar, Germany) using phase contrast with 40- and 100-fold magnifications. The images were transferred to the computer using the LEICA software (LAS X). The cell material was examined for hyphae, spores, and sporulating organs.

#### 2.3.2. Cell Lysis and Polymerase Chain Reaction (PCR)

For strain identification, the malt agar plates were inoculated with the respective fungal strain using the three-point inoculation technique and incubated at 30 °C for seven days. A small amount of newly grown cell material was then transferred with sterile forceps to a PCR tube. For each strain and primer pair, two biological replicates were performed. The cell lysis was performed according to the instructions in the DNeasy^®^ PowerSoil^®^ Kit (Qiagen, Hilden, Germany). The cells were lysed in the 3rd and 4th steps using the FastPrep-24 5G (MP Biomedicals, Santa Ana, CA, USA) with the QuickPrep adapter for 45 s at a speed of 5 m/s.

The isolated DNA was characterized by internal transcribed spacer (ITS) gene sequence analyses as described previously [31]. Fungal ITS regions were amplified using 1 µL DNA extract (cell material of one colony in 20 µL ddH_2_O) as template with oligonucleotides ITS1 (5′-TCCGTAGGTGAACCTGCGG-3′, 0.5 µM) and ITS4 (5′-TCCTCCGCTTATTGATATGC-3′, 0.5 µM) as primers [32]. Further strain identification using small subunit (SSU) 18S rRNA primers was performed using the primer pair (5′-AATTTGACTCAACRCGGG-3′; and 5′-GRGCATCACAGACCTG-3′, 0.5 µM each [33,34]).

The quality of the PCR reactions and the amplicons was checked using a 1% *w*/*v* agarose gel and a 1× TAE buffer. The gel was mixed with a staining agent (Roti^®^-GelStain, Carl Roth + Co. KG, Karlsruhe, Germany) and poured into an electrophoresis chamber (Biometra, Analytik Jena, Jena, Germany). The PCR products (2 µL) and 1 µL of ZR 50 bp DNA marker (Ready-to-Load, Zymo Research Europe, Freiburg im Breisgau, Germany) were tested using the electrophoresis chamber connected to a transformer (EV2310, Consort bvba, B-2300 Turnhout), and a voltage of 100 V was applied. After approximately 30 min, the gel was evaluated using the BDAdigital gel documentation system and BioDocAnalyze software version 2.66.3.44 (Biometra, Analytik Jena, Jena, Germany).

PCR products were purified using the DNA Clean & Concentrator Kit (Zymo Research D4003, Zymo Research Europe, Germany) according to the manufacturer’s instructions. The DNA concentration of the purified PCR products was determined using a spectrophotometer (DS-11+ Spectrophotometer, DeNovix, USA-19810 Wilmington). The spectrophotometer was first set up with 1 µL of DEPC-H2O.

Sanger sequencing was performed by Eurofins Genomics (Konstanz, Germany) with ITS1 and ITS4 and SSU-18S primers. The resulting forward and reverse sequences were assembled using the program Geneious (Geneious, Boston, MA, USA). The ITS sequences were compared with the NCBI ITS database using the Basic Local Alignment Search Tool (BLAST) algorithm (https://www.ncbi.nlm.nih.gov/, accessed on 20 December 2021 [35]) and with the Mycobank ITS database (https://www.mycobank.org/Pairwise_alignment/, accessed on 22 August 2023). The 18S sequences were compared with the NCBI SSU database.

### 2.4. Growth Experiment on Substrates

MSMHe plates (Section 2.1) were used for the growth experiments. The plates were inoculated with three 1 × 1 cm pieces of grown malt agar using the three-point method. For the fungi *P. javanicum* SBUG-M1741, *P. javanicum* SBUG-M1742, and *S. boydii* SBUG-M1749, 1 mL of tetradecane was pipetted onto a sterile filter paper disc (Whatman^®^, 110 mm ø, Hangzhou, China) in the lid of the glass petri dish. After five days of incubation, an additional 0.5 mL of substrate was added to the filter paper disc to avoid drying. For the fungi *F. oxysporum* SBUG-M1747 and *P. lilacinum* SBUG-M1751, 0.2 mL of pristane was pipetted onto the filter paper disc at the beginning and on the fifth day of the growth experiment. No substrate was added to the control plates to distinguish possible biomass growth on the plates with substrate. The inoculated plates with substrates were placed in glass incubation chambers, one glass incubation chamber for each substrate. The control plates were placed in a separate incubation chamber. The plates were incubated for at least seven days at 30 °C.

### 2.5. Biotransformation

#### 2.5.1. Cultivation

Malt broth (100 mL, Section 2.1) was added to a sterile 500 mL wide-neck flask closed with a sterile cotton plug. The malt broth was then inoculated with three 1 × 1 cm agar pieces from a 7-day-old grown malt agar plate. The cells were cultured for 7 days at 30 °C in a rotary shaker (VKS-75 CONTROL, Edmund Bühler, Bodelshausen, Germany) at 130 rpm. After cultivation, the medium and the filamentous fungal growth were homogenized using an Ultra-turrax homogenizer (IKA, Staufen, Germany) for three 10 s intervals at 13,000 rpm.

The obtained homogenate (10 mL) was transferred to 90 mL clean medium for incubation. As a control for determining the initial biomass, 10 mL of the homogenate was filtered, weighed after drying for 24 h at 100 °C.

#### 2.5.2. Incubation

For incubation with substrates, sterile 500 mL wide-neck flasks filled with MSMHe medium and supplemented with vitamins (Section 2.1) were used.

The sterile substrates were added to the flasks approximately 1 h before the start of the incubation. These flasks were shaken frequently to achieve good substrate distribution in the medium. The fungal homogenate (Section 2.5.1) was added to the medium. Two flasks were used as cell controls, containing 90 mL of MSMHe and 10 mL of the fungal homogenate without substrate. Two other flasks were used as substrate controls containing 100 mL of MSMHe but no fungal homogenate. In biotransformation experiments with tetradecane as substrate, 0.5 mL tetradecane was pipetted into the MSMHe medium of four flasks (0.5 mL per 100 mL incubation mixture in each of the four 500 mL flasks). For the incubation with pristane, 0.1 mL of pristane was pipetted into the MSMHe medium of four flasks (0.1 mL per 100 mL incubation mixture in each of the four 500 mL flasks). Biotransformation experiments with phytane included 0.01 mL of phytane added to the MSMHe medium of four flasks (0.01 mL per 100 mL incubation mixture in each of the four 500 mL flasks).

All flasks were incubated for 7 days at 30 °C. Four flasks of every substrate and every fungal strain (one cell control, one substrate control, and two biotransformation samples) were shaken in a rotary shaker at 130 rpm (shaking culture). The same sets of four flasks were incubated without shaking (static culture). At the end of the incubation, the dry weights of the biotransformation samples and cell controls were determined (Section 2.5.3), and the filtered supernatant was transferred to wide-neck flasks.

#### 2.5.3. Determination of Dry Weight

For the determination of dry weight, the glass-fiber filters (Whatman G6, Glass Fibre Filters, Diameter 50 mm) were first dried in an oven (Memmert + Co. KG, Schwabach, Germany) for 3 h at approximately 100 °C, weighed with a precision balance (OHAUS Europe, Greifense, Switzerland), and stored in a glass petri dish. A certain volume of the cell suspension was then filtered through the dried filters under vacuum. For the determination of the initial cell weight, 5 mL of the homogenate and the entire biotransformation samples were filtered. The filters were then dried for 24 h at approximately 100 °C, weighed with the precision balance, and the weight difference between the filter weight with biomass and the weight of an empty filter yielded the weight of the biomass.

### 2.6. Liquid–Liquid Extraction

The cell-free supernatants in the round-bottom flask were adjusted to a pH of 9.0 using 25% NaOH (Carl Roth + Co. KG, Karlsruhe, Germany). Diethyl ether (50 mL, Carl Roth + Co., KG, Karlsruhe, Germany) was used to extract the cell-free supernatant by shaking 3 times (5 min each) in a separating funnel. The organic solvent phases were combined and dried on anhydrous sodium sulfate (Merck, Darmstadt, Germany). The extract was then concentrated in a vacuum rotary evaporator (BÜCHI Labortechnik AG, Flawil, Switzerland) at 30 °C and approximately 650 mbar to a residual volume of approximately 1 mL. Evaporation to dryness was carried out under nitrogen gas. Residues obtained from biotransformation experiments were dissolved in 300 µL of methanol (Sigma-Aldrich, Taufkirchen, Germany), while those obtained from substrate controls were dissolved in 300 µL of hexane (Merck, Darmstadt, Germany). The remaining aqueous phase after removing the diethyl ether was adjusted to a pH of 2.0 using a 32% HCl solution and extracted with diethyl ether 3 times (5 min each). The obtained residue after drying was dissolved as mentioned above. All extracts were stored at approximately 4 ± 5 °C until measurement.

### 2.7. Methylation and Gas Chromatography–Mass Spectrometry (GC-MS)

The samples extracted in the acidic pH range were methylated prior to GC-MS analysis. For this purpose, 2 mL of 40% KOH (Carl Roth + Co. KG, Karlsruhe, Germany), 1 mL of carbitol, 200 mg diazald (Sigma-Aldrich, Taufkirchen, Germany), and 1 mL diethyl ether were sequentially added to a reaction apparatus with a side arm. The lid of the reaction apparatus was tightly closed, a Pasteur pipette was connected to the side arm, and it was held in the sample. The formed diazomethane could then enter the sample and cause the conversion of carboxyl and hydroxyl groups to methyl esters or methyl ethers, respectively. The extracts were evaporated overnight at room temperature.

GC-MS was used for the identification of metabolites. The extracts were first filtered. Then, the samples extracted in the acidic pH range were diluted 1:10 and those extracted in the alkaline pH range were diluted 1:100. The results of the measurement were then compared with the data from the NIST database based on the molecular weight and retention time to determine the metabolites. The configuration of the GC-MS system was according to the previously described method [27] Table S1.

## 3. Results

### 3.1. Identification of the Examined Fungal Strains

The ITS region of each strain was amplified and sequenced to identify the filamentous fungi under investigation. Comparing the sequencing data using BLAST tool at NCBI and Mycobank ITS databases has identified SBUG-M1741 and 1742 strains as *Penicillium javanicum*, SBUG-M1747 as *Fusarium oxysporum*, SBUG-M1749 as *Scedosporium boydii*, and SBUG-M1751 as *Purpureocillium javanicum* (Table 1) with identification details listed in Supplementary Data (Tables S2 and S3). Fungal ITS sequences were deposited in GenBank under the accession numbers listed in Table 1. Furthermore, the sequence information obtained after using SSU-18S primers was useful for the genus and, to some extent, the species determinations (Table S4). Parallel to the molecular identification of the strains, the microscopic examination helped to support the same finding. *P. javanicum* SBUG-M1741 sporulated on the entire 7-day-old MAg plate. Near the agar blocks, the strain grew light green to white, but turned greenish yellow to light brown at the mycelial edge (Figure S1a). The typical phialides for the strain were visible (Figure S1b), which resembled small brushes together with their conidia. *P. javanicum* SBUG-M1742 formed a plate mycelium around the agar blocks, which was pure white and powdery in appearance (Figure S1c). Spores were visible starting from day 7 of the growth and several phialides with a maximum of one spore were observed on the hyphae (Figure S1d). *S. boydii* SBUG-M1749 grew around the agar blocks in the form of a pure-white plate mycelium, with the mycelium lying soft and airy (Figure S1e). Typical for *Scedosporium* many hyphae and elongated spores were observed (Figure S1f). *F. oxysporum* SBUG-M1747 grew pinkish on the surface around the agar blocks. The strain could also grow into the agar (Figure S1g). Many hyphae, elongated spores, and phialides in the typical shape of *Fusarium* species were visible (Figure S1h). *P. lilacinum* SBUG-M1751 growth was cloud-like around the agar blocks but had a solid defined structure. The mycelium was light pink to purple (Figure S1i). The typical lemon-shaped spores were microscopically visible. In addition, the head of a phialide with spores and the chlamydospore could also be detected (Figure S1j,k).

### 3.2. Ability of the Tested Fungi to Use Oil Components as Growth Substrates

The strains *P. javanicum* SBUG-M1741 and SBUG-M1742 and *S. boydii* SBUG-M1749 were originally isolated on tetradecane, while *F. oxysporum* SBUG-M 1747 and *P. lilacinum* SBUG-M1751 on pristane; therefore, these oil components were used as growth substrates.

#### 3.2.1. Growth on Tetradecane

After five days of incubation, strong growth was observed for *P. javanicum* SBUG-M1741 and SBUG-M1742. Plates with tetradecane as the sole source of carbon and energy showed further growth after 7 days compared to the control plates without a carbon source, which showed weak or no growth (Table S5). The growth experiments with their replicates suggest that both *P. javanicum* strains can use tetradecane as the sole carbon and energy source. Unlike the *P. javanicum* strains, the growth of *S. boydii* SBUG-M1749 on MSMH plates containing tetradecane was similar to the control (Table S5). After extending the experiment to 20 days, no further growth was observed, suggesting that tetradecane is neither a good carbon source nor toxic for *S. boydii*.

#### 3.2.2. Growth on Pristane

*F. oxysporum* SBUG-M1747 showed weak growth in the presence or absence of pristane, indicating that pristane is not a good carbon source for SBUG-M1747 until 20 days of growth (Table S6). Similarly, the growth of *P. lilacinum* SBUG-M1751 on plates with pristane was very comparable to the control plates (Table S6). However, after 20 days of growth the control plates had exhibited more growth than the plates containing pristane, indicating a possible growth inhibition effect of the substrate after longer incubation period.

### 3.3. Biodegradation of Oil Components as Substrates by the Tested Fungal Strains

Filamentous fungi were tested for their ability to use and degrade tetradecane, pristane, and phytane as model substrates of aliphatic oil components. For this purpose, a pre-culture was first prepared, and the obtained cell suspension was transferred to the incubation approaches with the respective substrate. After seven incubation days, the change in the biomass was determined to estimate the fungal growth. The obtained pH 9 and pH 2 extracts (Section 2.6) were processed and analyzed using GC-MS (Table S1, Section 2.7). Subsequently, the chromatograms were investigated for the remaining substrate content and possible degradation products.

The strains *P. javanicum* SBUG-M1741, SBUG-M1742 and *S. boydii* SBUG-M1749 originally isolated on tetradecane were used with this substrate. *F. oxysporum* SBUG-M 1747 and *P. lilacinum* SBUG-M1751 originally isolated on pristane were used with this isolation substrate. Strains with particularly good degradation results on the isolation substrate were also tested on additional substrates—*P. javanicum* SBUG-M1741 on pristane, *P. lilacinum* SBUG-M1751 on tetradecane and phytane.

#### 3.3.1. Biodegradation of Tetradecane and Pristane by *P. javanicum*

**Figure 1:**
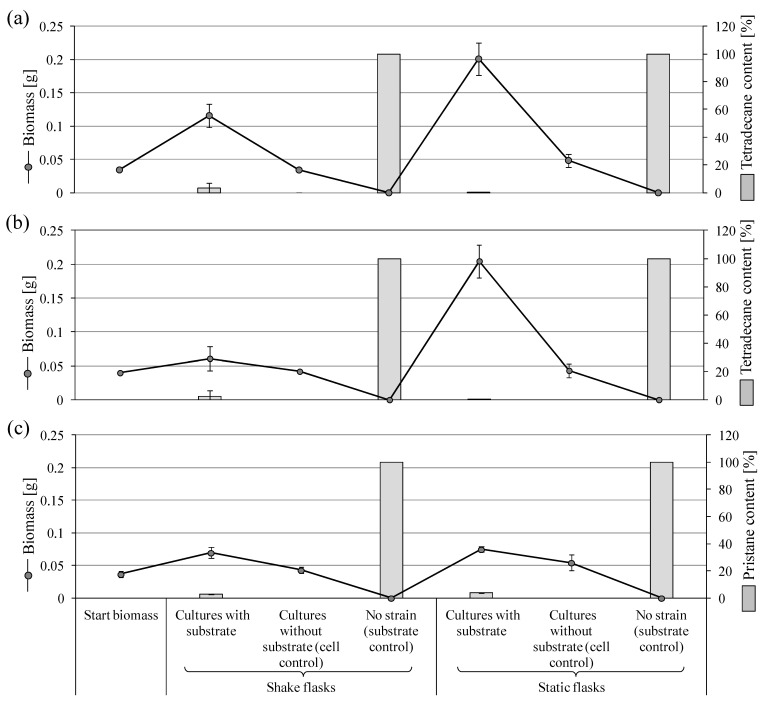
Growth of *P. javanicum* strains on tetradecane or pristane as the sole carbon and energy source and the remaining substrate after 7 days of incubation. (**a**) SBUG-M1741 with 0.5% tetradecane, (**b**) SBUG-M1742 with 0.5% tetradecane, and (**c**) SBUG-M1741 with 0.1% pristane.

At the end of the incubation of *P. javanicum* SBUG-M1741 with tetradecane, many small cream-colored pellets were visible in the shake culture approaches, whereas in the cell control, diffuse light cream-colored material of different sizes was visible. Biotransformation experiment within the shake culture approaches had a biomass growth equivalent to 3.4 times the starting biomass, whereas there was no biomass increase in the absence of tetradecane (cell control; Figure 1a). The static cultures with tetradecane showed that a 1 cm thick mycelial plate formed on the surface, which had a green to white color and was significantly thicker than that of the cell control. The cell control was slightly covered with mycelium on the surface and had a lighter color than the biotransformation approaches. Biotransformation experiments of the static cultures had a cell biomass of about four times the cell control and six times the starting biomass (Figure 1a). *P. javanicum* SBUG-M1741 was able to significantly degrade tetradecane by ≥97% in both shake and static flasks. The strain could use this substrate as an energy and carbon source, which was also evident from the significant increase in the biomass (Figure 1a).

Similar to *P. javanicum* SBUG-M1741, the *P. javanicum* SBUG-M1742 strain was also able to grow on tetradecane (Figure 1b). Analyses of the extracts of both *P. javanicum* strains confirmed the formation of different metabolites in both shake and static flask approaches of the acidic extracts (Table 2), whereas no degradation products were detected in alkaline extracts. *P. javanicum* SBUG-M1741 formed seven detectable metabolites in both shake and static approaches. Four out of six metabolites could be detected in the extracts prepared from *P. javanicum* SBUG-M1742 shake and static cultures (i.e., tetradecanoic, dodecanoic, octanoic, and hexanoic acids), while extracts prepared from shake cultures showed an additional two metabolites (i.e., hexanedioic and decanoic acids; Table 2).

Since *P. javanicum* SBUG-M1741 showed optimal ability to degrade tetradecane, it was interesting to know whether it is also capable of degrading branched-chain alkanes. Therefore, a conversion experiment with pristane at a starting concentration of 0.1% was carried out. The biotransformation experiments from the shake flask approaches had doubled the biomass (Figure 1c) and showed many small cream-colored pellets. The shake cell control formed longer threads with an unappreciable biomass increase compared to the starting biomass. The biomass growth of the static biotransformation approaches was comparable to the shake flask cultures. In static flasks, a very fine mycelial plate had formed on the surface with diffused material in the medium. The mycelial plate of the cell control from the static flasks had a biomass increase of 0.0177 g compared to the starting biomass, which was significantly lower than the increase in the static biotransformation approaches when pristane is included (i.e., 0.038 g) but 1.26 times the cell control biomass in shake flasks. The degradation of pristane by *P. javanicum* SBUG-M1741 was almost complete. A degradation of more than 97% was detected in the biotransformation approaches from the shake flask cultures. The biotransformation approaches of the static cultures had a slightly lower degradation of 96% (Figure 1c).

The degradation experiment of pristane by *P. javanicum* SBUG-M1741 showed the formation of three metabolites. These were exclusively present in the biotransformation approaches of the shake flask cultures (Table 3).

Overall, *P. javanicum* SBUG-M1741 was able to degrade both the un-branched (i.e., tetradecane) and the simple-branched alkane (i.e., pristane). When tetradecane was used as a substrate, the biomass content of the static cultures was higher than the shake cultures (Figure 1a,b). Nonetheless, a comparable increase in the biomass was observed in these cultures when incubated with pristane (Figure 1c). Generally, more acids were detected in the shake flask cultures (Table 2 and Table 3).

#### 3.3.2. Degradation of Tetradecane by *S. boydii* SBUG-M1749

**Figure 2:**
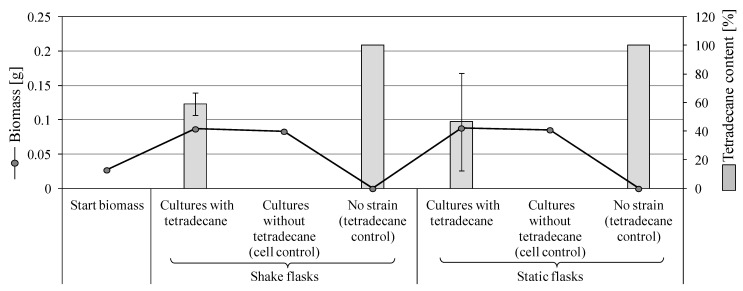
Growth of *S. boydii* SBUG-M1749 and the remained substrate after 7-day incubation with 0.5% tetradecane as the sole carbon and energy source.

No appreciable difference in the growth was observed among shake and static cultures of *S. boydii* strain incubated with tetradecane. Macroscopically, several diffused gray-brown clumps and long threads of different sizes were formed in shake incubations, while the static cultures showed a fine translucent white mycelial plate with diffuse, cream-white clumps in the medium. In the cell control, an opaque white mycelial plate was observed, keeping the medium similar to the biotransformation approaches and with comparable growth (Figure 2). Shake and static cultures with tetradecane exhibited around 3.2 times biomass increase compared to the starting biomass (Figure 2). However, this increase was comparable to the corresponding control cultures without tetradecane, which supports the aforementioned poor growth of *S. boydii* plates with tetradecane (Section 3.2.1, Table S5). At the end of the incubation period, around 59 and 46% of tetradecane remained un-degraded in shake and static biotransformations, respectively (Figure 2). *S. boydii* SBUG-M1749 showed lower tetradecane degradation compared to *P. javanicum* strains (Figure 1a,b). Interestingly, at lower tetradecane concentration (0.25%), *S. boydii* could efficiently degrade the substrate (i.e., >98%), albeit with no visible difference in the biomass increase.

In total, six metabolites were formed after incubation with *S. boydii* SBUG-M1749 strain including hexanedioic acid. There were no significant differences between the cultivation methods (static and shake flasks) except for hexanoic acid, which was only found in the shake flask approach (Table 2).

#### 3.3.3. Degradation of Pristane by *F. oxysporum* SBUG-M1747

**Figure 3:**
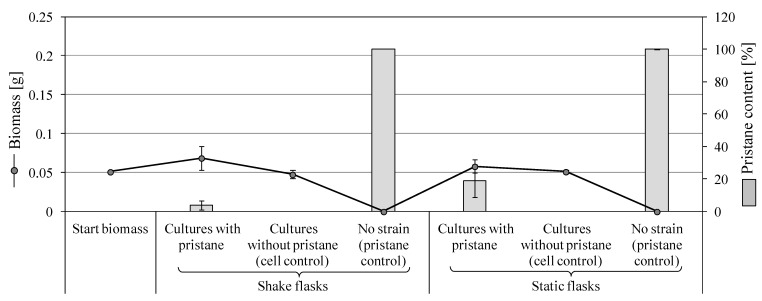
Growth of *F. oxysporum* SBUG-M1747 and the remaining substrate after 7-day incubation with 0.1% pristane as the sole carbon and energy source.

Diffused cell clumps of *F. oxysporum* with an increase in the shake culture biomass by 34.7% were observed in the presence of pristane. Cell cultures with no pristane (cell controls) formed rod-shaped cells and experienced biomass reduction of 6.6% compared to the starting biomass. Static cultures showed fine light-yellow mycelium on the surface that changed to light pink at the edges. Compared to the starting biomass, unappreciable increase of 13.6% was observed in the presence of pristane. Correspondingly, no change in the starting biomass of the cell controls in the absence of pristane was detected (Figure 3). This result is consistent with the observed weak growth (Section 3.2.2, Table S6). GC-MS analyses confirmed the biotransformation of pristane, which was higher in shake than static culture approaches (96.2% vs. 81%; Figure 3). A dicarboxylic acid, 2-methylpentanedioic acid, was also detected (Table 3).

#### 3.3.4. Biodegradation of Tetradecane, Pristane, and Phytane by *P. lilacinum* SBUG-M1751

**Figure 4:**
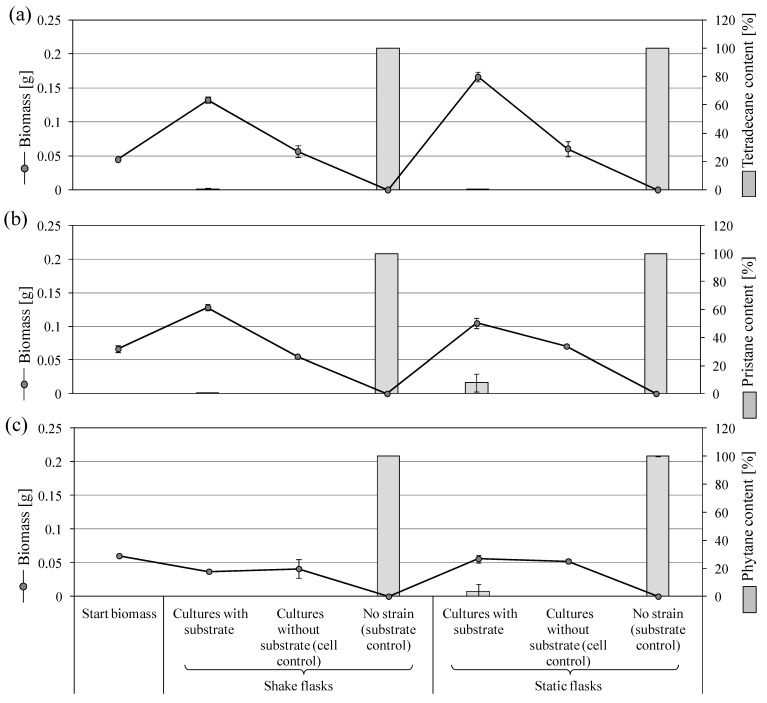
Growth of *P. lilacinum* SBUG-M1751 on tetradecane, pristane, or phytane as the sole carbon and energy source and the remaining substrates after 7 days of incubation. (**a**) 0.25% tetradecane, (**b**) 0.1% pristane, and (**c**) 0.01% phytane.

*P. lilacinum* SBUG-M1751 was incubated with 0.25% tetradecane as an unbranched *n*-alkane. Many small, cream-colored pellets were observed in shake cultures which exhibited a three-fold increase in biomass. No visible increase in the biomass was recorded in the absence of tetradecane (cell controls of shake cultures). The static flask approaches formed a white to pink mycelial plate at the surface. In the cell control, this mycelial plate was not as densely grown as in the biotransformation approaches. At the end of the incubation period, the biomass of the static cultures incubated with tetradecane was 1.2 times the biomass of the shake flask counterparts (Figure 4a).

Tetradecane degradation by *P. lilacinum* SBUG-M1751 was almost complete (Figure 4a). The strain bioconverted tetradecane into five mono-carboxylic acids and one di-carboxylic acid (Table 2). The hexanoic and hexanedioic acid could only be detected in the biotransformation approach of the static culture. All other carboxylic acids could be regularly detected in each biotransformation approach.

Bioconversion experiments of pristane by *P. lilacinum* showed diffuse, yellow-brown cell material accompanied by doubling in the biomass of the shake cultures, while the cell control of the shake flask culture showed no further growth. The static flask approaches turned cloudy white to yellow in color. A fine mycelium grew on the surface, which had the same medium color, but with more diffuse cream-white cell clumps. Cultures without pristane (cell controls) showed no appreciable increase in the biomass, while the presence of pristane caused a weak biomass increase of 57.5% compared to the starting biomass (Figure 4b). More than 90% degradation was observed in shake and static biotransformation approaches with better degradation in shake cultures (Figure 4b). In the incubation approach with pristane and *P. lilacinum*, five dicarboxylic acids could be detected (Table 3).

Biodegradation experiments with phytane showed diffused cell clumps. Cloudy medium with fine mycelium plate growing on the surface was additionally observed in the static cultures. A decrease to no change in biomass was measured in shake and static incubations. This suggests that phytane might have a toxic effect and thus inhibited this strain from growing compared to controls (Figure 4c). The biotransformation approaches showed proper degradation of phytane (96–100%; Figure 4c). Extract analyses showed three carboxylic acids, two of which (i.e., 3-methylhexanedioic acid and 4-methylpentanoic acid) were exclusively formed in shake cultures (Table 4).

Finally, it can be summarized that *P. javanicum* SBUG-M1741 showed the best results in the degradation of tetradecane compared to SBUG-M1742 and *S. boydii* SBUG-M1749 strains due to the decrease in the remained tetradecane and the number of products formed. For this reason, *P. javanicum* SBUG-M1741 was also tested for the utilization of pristane. Comparatively, visible pristane degradation ability could also be demonstrated for this strain. However, the conversion was not quite as good as that of *P. lilacinum* SBUG-M1751 on pristane, but considerably better than that of *F. oxysporum* SBUG-M 1747 on pristane, although these two strains were isolated on pristane and *P. javanicum* SBUG-M1741 on tetradecane. When compared to *F. oxysporum* SBUG-M 1747, *P. lilacinum* SBUG-M1751 showed better conversion of pristane. Thus, the degradation experiments of the *P. lilacinum* strain were extended to include tetradecane and phytane as substrates, showing acceptable utilization potential for these two substrates. It is worth noting that the degradation of tetradecane by *P. lilacinum* SBUG-M1751 is comparable to that of *P. javanicum* SBUG-M1741.

## 4. Discussion

The bioremediation of oil pollutants had been previously investigated using various microorganisms like bacteria, fungi, and algae [36,37,38]. However, the exceptional metabolic abilities of filamentous fungi suggest them as a secure and sustainable eco-friendly and cost-effective tool in the bioremediation of oil hydrocarbons. These properties inspired the researchers to screen the substrate specificity of more strains and cover complex hydrocarbons [39,40]. Within the same scenario, oil-polluted soil samples were targeted as the native habitat of the five fungal isolates examined in this study. Here, the use of these strains was not limited to examining their potential bioremediation ability at laboratory level, but also as a promising tool to relief eco-systems from crude oil spills by re-introducing these fungal strains into their contaminated, but native, habitats. Fungal-based remediation can include non-pathogenic and pathogenic strains. In this article, two *Penicillium* isolates were used as examples for non-pathogenic fungi, while *Scedosporium*, *Purpureocillium*, and *Fusarium* species represent opportunistic and common human pathogens.

### 4.1. Degradation Potential of Tetradecane

Tetradecane is a model compound for *n*-alkanes, which are always present in crude oil. Its degradation can follow mono- and/or di-terminal oxidation mechanisms [17,41,42,43]. In this work, *Penicillium javanicum* SBUG-M1741 and *Scedosporium boydii* SBUG-M1749 exhibited both degradation mechanisms presented by the formation of mono- and di-carboxylic acids, while only mono-terminally oxidized products were detected in the extracts prepared from *P. javanicum* SBUG-M1742 and *Purpureocillium lilacinum* SBUG-M1751 biotransformation cultures.

Depending on the chain length of the substrate, different enzymes could be integrated into the process; C_1_–C_4_ are known to be oxidized by the methane monooxygenase and C_5_ to C_16_ by alkane hydroxylases or cytochrome P450 monooxygenases [16]. Recently, *Aspergillus flavus* monooxygenases have been identified as potential bricks in the degradation of long-chain alkanes (>C_16_; [44]). The genus *Penicillium* isolated from oil-contaminated soil has been described as the most active oil degrader [45,46,47]. The same held true for *P. javanicum* strains in this study, showing an exclusive degradation ability of tetradecane. In a study by Oudot et al. [45], a turnover of crude oil with 10% was described for *P. citrinum*. Another study suggested that *P. pinophilum*, *P. brevicompactum*, and *P. simplicissimum* degraded saturated hydrocarbons from 46 to 55%, but aromatic hydrocarbons only from 10 to 38% [46]. Recently, the degradation of *n*-alkanes by *P. lilacinum* was 21% and that of *iso*-alkanes was 14.5%, but cycloalkanes and aromatics were used by 100% and 15.1% [24], respectively. In the present work, *P. javanicum* and *P. lilacinum* strains were able to degrade tetradecane efficiently (turnover > 96%) with visible growth, especially in the static flask approaches. Similar remarkable biomass increase could also be demonstrated for *P. chrysogenum* and *P. citrinum* grown on crude oil and *n*-alkanes (C_13_–C_18_) [47,48].

The current results demonstrate that *S. boydii* could presumably degrade tetradecane but cannot use it for remarkable growth. In a study by Yuan et al. [49], an indigenous bacterial consortium, which originated from an oil-contaminated soil, could degrade crude oil more effectively in a co-culture with *S. boydii*. Thus, the degradation of crude oil by the bacterial consortium could be increased from 61.06% to 81.45% in co-culture with *S. boydii*, whereas *S. boydii* alone used only 30% [49]. In the current work, *S. boydii* could degrade tetradecane to 46.6% in static cultures. This suggests that *S. boydii* may not show outstanding potential as a sole crude oil or *n*-alkanes degrader. Nevertheless, *S. boydii* could increase the degradation potential of these pollutants when co-cultured with bacteria, because it was probably able to convert components of the crude oil from toxic to less toxic or non-toxic products [49], which in turn are better utilizable by the bacteria. Similarly, a recent study has examined an increase in the degradation rate of total petroleum hydrocarbon by *Scedosporium* strain in the presence of *Acinetobacter* sp. from 23.36% to 58.61% [50].

**Figure 5:**
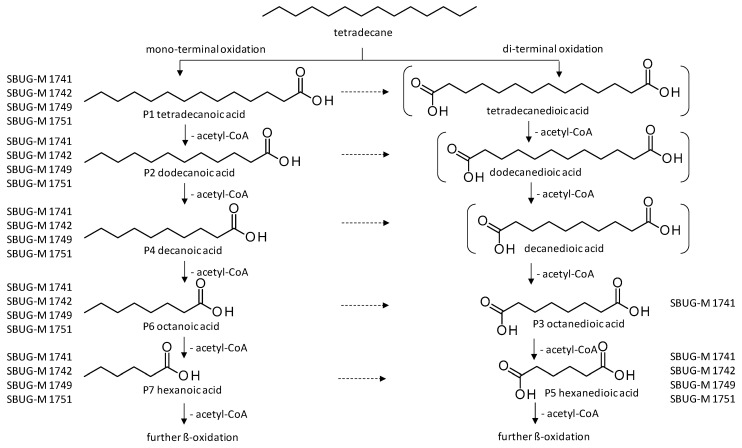
Degradation scheme of the carboxylic acids after methylation presenting mono- and di-terminal oxidations of tetradecane by *Penicillium javanicum* SBUG-M1741 and SBUG-M1742, *Scedosporium boydii* SBUG-M1749, and *Purpureocillium lilacinum* SBUG-M1751. Structures that were not detected in the current study are marked by brackets. Pn refers to the product number as per its appearance in Table 2.

By the GC-MS analyses, both mono- and dicarboxylic acids could be detected for the four strains *P. javanicum* SBUG-M1741 and SBUG-M1742, *S. boydii* SBUG-M1749, and *P. lilacinum* SBUG-M1751 as intermediates in the degradation of tetradecane. Due to the detection of octane- and/or hexanedioic acids (P3, P5; Figure 5), a di-terminal degradation of the tetradecane can be assumed for these strains. Furthermore, for all of these strains, the mono-terminal degradation by all possible monocarboxylic acids starting from tetradecanoic (P1) acid to hexanoic acid (P7) could be proven. The efficient degradation of tetradecane by *P. lilacinum* has been previously confirmed together with the formation of mono- and di-carboxylic acids [31].

### 4.2. Degradation Potential of Pristane

*P. lilacinum* SBUG-M1751 showed weak biomass formation, but visible degradation of pristane (>90%). The same results of *P. lilacinum* had been previously observed when heating oil was used as substrate, albeit with no further study on the degradation products [48]. Very recently, *P. lilacinum* isolated from heavy oil sludge (north China) showed 100%, 21.2%, 15.1%, and 14.5% degradation within 30 days for cycloalkanes, *n*-alkanes, aromatics, and *iso*-alkanes, respectively [24]. Unlike *P. lilacinum* SBUG-M1751, *P. javanicum* SBUG-M1741 was able to grow remarkably on pristane with higher degradation ability (>97%). Similar growth was also reported by two bacterial strains: *Mycobacterium neoaurum* and *Rhodococcus ruber* [51]. The reported degradability of pristane by bacterial marine populations is variable (>80%; [52]), while that with *Rhodococcus* sp. is about 40% [53].

**Figure 6:**
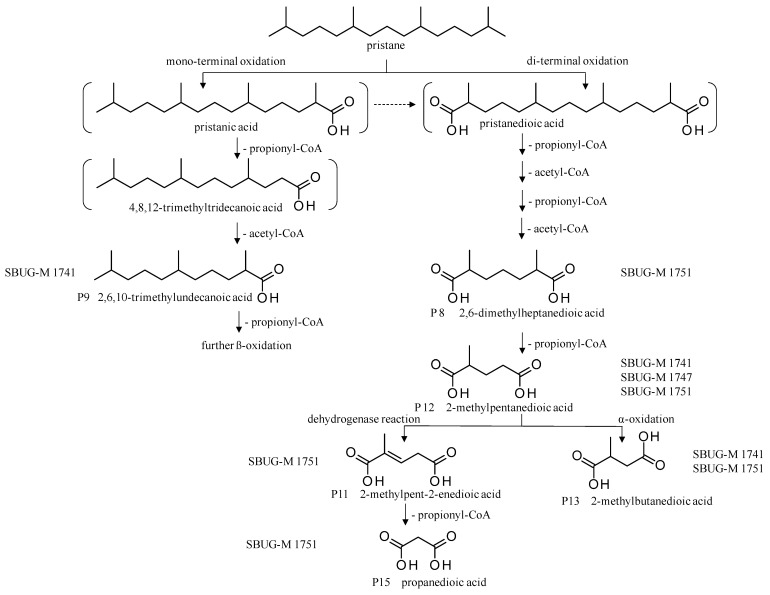
Degradation scheme of the carboxylic acids after methylation presenting mono- and di-terminal oxidations of pristane by *Penicillium javanicum* SBUG-M1741, *Fusarium oxysporum* SBUG-M1747, and *Purpureocillium lilacinum* SBUG-M1751 [51,56]. Structures that were not detected in the current study are marked by brackets. Pn refers to the product number as per its appearance in Table 3.

Pristane degradation by *F. oxysporum* SBUG-M1747 was highest in the shake flask approaches (~96%). In a study by Simister et al. [54], *F. solani* showed a degradation of ~35%, 41%, and 97% as turnover values with alkanes (C_19_–C_36_), crude oil, and alkanes (C_11_–C_18_), respectively. Recently, and for the first time, the oil-degrading ability of three *Fusarium* isolates from oil tankers (Saudia Arabia) was confirmed. Neither the degradation mechanisms nor products were investigated as the tested substrates were a heterogeneous mixture of oils including crude oil, used oil, diesel, and kerosene [55]. In the current work, the degradation of pristane suggests that only *P. javanicum* SBUG-M1741 is able to apply mono- and di-terminal oxidation based on the detection of the monocarboxylic acid 2,6,10-trimethylundecanoic acid (P9) and the dicarboxylic acids 2-methylpentanedioic (P12) and 2-methylbutanedioic acid (P13; Figure 6). Similarly, the bacterial degradation of pristane follows both oxidation mechanisms [51,52]. *F. oxysporum* SBUG-M1747 and *P. lilacinum* SBUG-M1751 followed presumably exclusively di-terminal oxidation based on the detection of different dicarboxylic acids (Figure 6). Based on the detection of 2-methylbutanedioic acid (P13) for *P. javanicum* SBUG-M1741 and *P. lilacinum* SBUG-M1751, it can be assumed that α-oxidation plays an important role in the degradation of pristane by these two strains in addition to β-oxidation.

The detailed biodegradation of pristane has been investigated and described in yeasts and bacteria [31,51,52,56,57].

### 4.3. Degradation Potential of Phytane

Phytane (C_20_) is a methyl-branched alkane that is structurally similar to pristane (C_19_), but it is characterized by a β-position branching point at one end of the molecule, which makes it more difficult to degrade because of the steric hindrance of β-oxidation enzymes.

**Figure 7:**
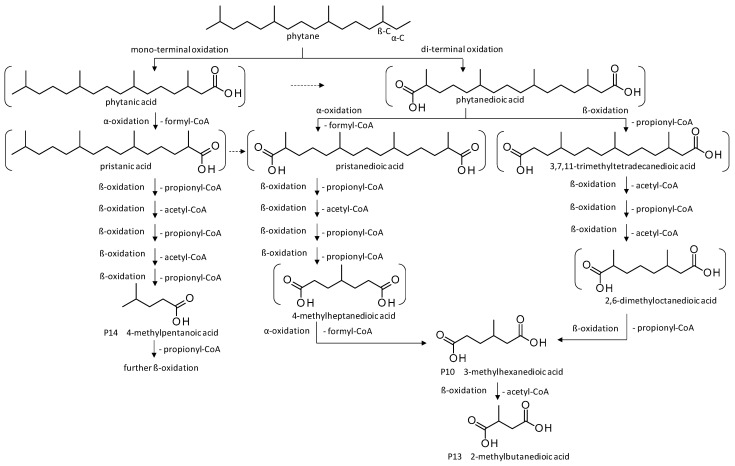
Degradation scheme of the carboxylic acids after methylation presenting mono- and di-terminal oxidations of phytane by *Purpureocillium lilacinum* SBUG-M1751. The α-oxidation is adapted according to previously published data [51,52,58]. Structures that were not detected in the current study are marked by brackets. Pn refers to the product number as per its appearance in Table 4.

*P. lilacinum* SBUG-M1751 showed no biomass growth on phytane, which could be due to its low available substrate concentration (i.e., 0.01%). Experiments with *Mycobacterium fortuitum* NF4 and *M. ratisbonense* SD4 showed good growth with 0.2% *v*/*v* phytane [58]. If a comparable concentration of phytane was considered in the current study, an appreciable biomass increase would probably also be measurable for *P. lilacinum* SBUG-M1751, but the cost–benefit consideration in purchasing a correspondingly large quantity of phytane showed that it is sufficient to determine the degradation of this substrate by means of substrate decrease and metabolite formation. Other bacteria had also shown potential toward the degradation of phytane [58,59]. In a study by Nakajima et al. [59], *Rhodococcus* sp. BPM 1613 was able to transform phytane into 2,6,10,14-tetramethyl-1-hexadecanol and 2,6,10,14-tetramethylhexadecanoic acid. Phytane was almost completely degraded by *P. lilacinum* SBUG-M1751 through mono- and di-terminal oxidation pathways based on the detection of 4-methylpentanoic (P14) acid as metabolite for the mono-terminal oxidation and 3-methylhexanedioic (P10) and 2-methylbutanedioic acid (P13) as intermediates for the di-terminal oxidation pathway (Figure 7). Furthermore, in addition to β-oxidation, α-oxidation had a key role in the degradation of phytane, since only α-oxidation steps can help to break down the β-branching point. Thus, starting from the initial mono-terminal attack with the formation of phytanic acid or from the initial di-terminal attack with the formation of phytanedioic acid, α-oxidation could take place in the very next step. Alternatively, α-oxidation processes take place in the further course of the degradation reactions. Similar α-oxidation steps were proposed for the di-terminal oxidation pathway for the degradation of phytane by the yeast *Moniliella spathulata* [57]. Furthermore, the biodegradability of pristane and phytane by fungi isolated from oil-soaked sand beach has been reported (70–98%, [54]).

## 5. Conclusions

These results of the isolated fungal strains show that they are able to degrade *n*-alkanes and branched-chain hydrocarbons efficiently. The degradation of tetradecane, pristane, and phytane as model substrates of crude oil components proceeds via mono- and also di-terminal oxidation mechanisms. The α- and ß-oxidation reaction steps are involved in these pathways. This offers an environmental remediation tool based on fungal strains for dealing with the serious impacts of petroleum hydrocarbons to restore ecosystems.

## Figures and Tables

**Table 1:** Strain identification based on similarity to publicly available ITS sequences.

Strain	GenBank Accession N° of Isolate	NCBI ITS Database			Mycobank ITS Database			
		Query Length (nt) *	Best Hit (Accession N°) **	Identity (%)	Best Hit (Description) ***	Score	Overlap (%)	Identity (%)
SBUG-M1741	OR335318	529	*Penicillium javanicum* (MH865296.1)	99.81	*Eupenicillium* javanicum **** (CBS 291.53)	835	100	99.81
SBUG-M1742	OR335319	521	*Penicillium javanicum* (MH865296.1)	99.81	*Eupenicillium javanicum* **** (CBS 291.53)	824	100	99.81
SBUG-M1747	OR335322	482	*Fusarium oxysporum* (MK074845.1)	100	*F. oxysporum* species complex (LC13769 MW016603)	765	100	100
SBUG-M1749	OR335324	552	*Scedosporium boydii* (KP132690.1)	100	*Scedosporium boydii* (CNRMA16.348)	876	100	100
SBUG-M1751	OR335323	569	*Purpureocillium lilacinum* (MH426603.1)	100	*Purpureocillium lilacinum* (CNRMA18.195)	901	99.82	100

* Query coverage was 100% for all blasted sequences with E value of 0. ** https://www.ncbi.nlm.nih.gov/, accessed on 15 June 2023; *** https://www.mycobank.org/Pairwise_alignment, accessed on 15 June 2023; **** Synonym for *Penicillium javanicum*.

**Table 2:** Detected acids formed during the degradation of tetradecane by *Penicillium javanicum* SBUG-M1741 and SBUG-M1742, *Scedosporium boydii* SBUG-M1749, and *Purpureocillium lilacinum* SBUG-M1751.

Products		Retention Time (min)	*P. javanicum*				*S. boydii* SBUG-M1749		*P. lilacinum* SBUG-M1751	
			SBUG-M1741		SBUG-M1742					
			Type of Culture							
			Shake	Static	Shake	Static	Shake	Static	Shake	Static
P1	Tetradecanoic acid 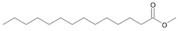	32.59	+	+	+	+	+	+	+	+
P2	Dodecanoic acid 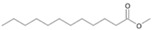	26.95	+	+	+	+	+	+	+	+
P3	Octanedioic acid 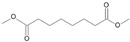	24.9	+	+	-	-	-	-	-	-
P4	Decanoic acid 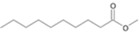	20.65	+	+	+	-	+	+	+	+
P5	Hexanedioic acid 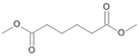	18.0	+	+	+	-	+	+	-	+
P6	Octanoic acid 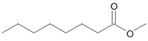	13.86	+	+	+	+	+	+	+	+
P7	Hexanoic acid 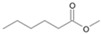	7.4	+	+	+	+	+	-	-	+

+, detectable; -, non-detectable.

**Table 3:** Detected acids formed during the degradation of pristane by *Penicillium javanicum* SBUG-M1741, *Fusarium oxysporum* SBUG-M1747, and *Purpureocillium lilacinum* SBUG-M 1751.

Products		Retention Time (min)	*P. javanicum* SBUG-M1741		*F. oxysporum* SBUG-M1747		*P. lilacinum* SBUG-M1751	
			Type of Cultures					
			Shake	Static	Shake	Static	Shake	Static
P8	2,6-Dimethylheptanedioic acid 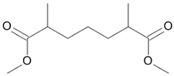	23.6	-	-	-	-	+	+
P9	2,6,10-Trimethylundecanoic acid 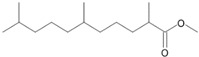	23.36	+	-	-	-	-	-
P10	3-Methylhexanedioic acid 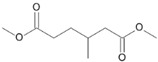	19.3	-	-	-	-	-	-
P11	2-Methylpent-2-enedioic acid 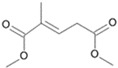	17.7	-	-	-	-	+	+
P12	2-Methylpentanedioic acid 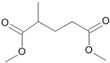	15.6	+	-	+	+	+	+
P13	2-Methylbutanedioic acid 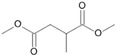	11.4	+	-	-	-	+	+
P14	4-Methylpentanoic acid 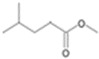	10.1	-	-	-	-	-	-
P15	Propanedioic acid 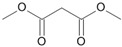	7.4	-	-	-	-	+	+

+, detectable; -, non-detectable.

**Table 4:** Detected acids formed during the degradation of phytane by *Purpureocillium lilacinum* SBUG-M1751.

Products		Retention Time (min)	*P. lilacinum* SBUG-M1751	
			Type of Cultures	
			Shake	Static
P10	3-Methylhexanedioic acid 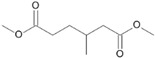	19.3	+	-
P13	2-Methylbutanedioic acid 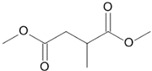	11.4	+	+
P14	4-Methylpentanoic acid 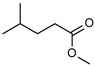	10.1	+	-

+, detectable; -, non-detectable.

## Data Availability

The data presented in this study are available within the article and Supplementary Materials.

## Supplementary Materials

The following supporting information can be downloaded at https://www.mdpi.com/article/10.3390/microorganisms11092195/s1. Figure S1: Macro- and microscopical views of filamentous fungi grown on MAg plates for 7 days; Table S1: GC-MS protocol; Table S2: Results of identification of the isolated filamentous fungi by ITS gene sequence analysis in NCBI ITS database; Table S3: Results of identification of the isolated filamentous fungi by ITS gene sequence analysis in Mycobank ITS database; Table S4: Results of identification of the isolated filamentous fungi by 18S gene sequence analysis in the NCBI SSU database; Table S5: Growth experiment of SBUG-M 1741, SBUG-M1742 and SBUG-M 1749 on tetradecane after 5 and 7 days; Table S6: Growth experiments of SBUG-M 1747 and SBUG-M 1751 on pristane after 5 and 7 days.
