# Supplementary material for: Characterization of the Mycoremediation of n-Alkanes and Branched-Chain Alkanes by Filamentous Fungi from Oil-Polluted Soil Samples in Kazakhstan
Source: Microorganisms. 2023 Aug 30;11(9):2195. doi: 10.3390/microorganisms11092195 (PMC10534712; doi:10.3390/microorganisms11092195)
Supplement: Supplementary file 1 [file microorganisms-11-02195-s001.zip › microorganisms-2536653-supplementary.pdf]

microorganisms

Supplementary materials

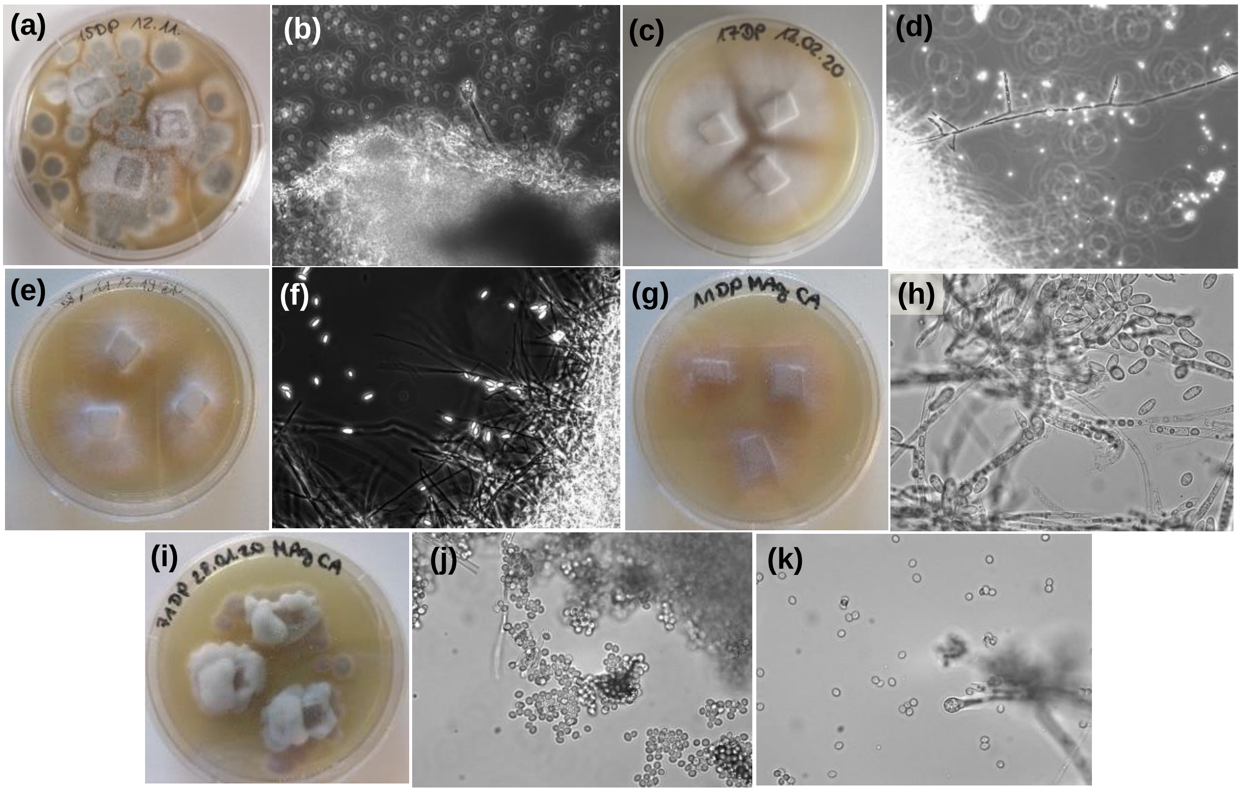

Figure S1. Macro-and microscopical views of filamentous fungi grown on MAg plates for 7 days. (a, b) *Penicillium javanicum* SBUG-M 1741 showing intact conidial carrier on a hypha and many spores in the background. (c, d) *Penicillium javanicum* SBUG-M 1742 with several phialides and some spores. (e, f) *Scedosporium boydii* SBUG-M 1749 with some spores and hyphae. (g, h) *Fusarium oxysporum* SBUG-M 1747 with spores, hyphae, and a phialide. (i, j, k) *Purpureocillium lilacinum* SBUG-M 1751 presenting spores, hyphae, phialide and a chlamydospore. Magnification power is 40x for b, d, f and 100x for h, j, k.

Table S1. GC-MS protocol.

**Gas-chromatography**	Agilent 7890A GC-System
Mass-spectrometer	
Automatic liquid sampler	Agilent 7693A ALS
Capillary column	HP-5MS Ultra Inert<br>30 m, 0,25 mm, 0.25 µm<br>5 % Diphenyl / 95 % Dimethylpolysiloxan
Carrier gas	Helium<br>Pressure: 11.731 psi<br>Flow: 1.2046 ml min^{-1}
Temperature programme 1<br>(for alkaline extracts)	0 min 40 °C; 40 – 310 °C: 8 °C min^{-1}; 6.25 min 310 °C
Temperature programme 2<br>(for acidic extracts)	0 min 40 °C; 40 – 310 °C: 4 °C min^{-1}; 7.5 min 310 °C
Ionization	70 eV
Interface temperature	240 °C
MS source temperature	250 °C
Injection volume	1 µL

Table S2. Results of identification of the isolated filamentous fungi by ITS gene sequence analysis in NCBI ITS database.

Isolate	Result of identification	ITS gene sequence analysis[a)]					
		Description	Max score	Total score	Query cover	Identity	Accession N°
**SBUG-M 1741 (15/P17/K2)**	*Penicillium javanicum*	*Penicillium javanicum* strain CBS 129395 small subunit ribosomal RNA gene, partial sequence; internal transcribed spacer 1, 5.8S ribosomal RNA gene, and internal transcribed spacer 2, complete sequence; and large subunit ribosomal RNA gene, partial sequence	972	972	100%	99.81%	MH865296.1
		Penicillium javanicum var. javanicum strain CBS 291.53 small subunit ribosomal RNA gene, partial sequence; internal transcribed spacer 1, 5.8S ribosomal RNA gene, and internal transcribed spacer 2, complete sequence; and large subunit ribosomal RNA gene, partial sequence	972	972	100%	99.81%	MH857207.1
		Eupenicillium sp. LG41 genomic DNA sequence contains ITS1, 5.8S rRNA gene and ITS2, isolate LG41	972	972	100%	99.81%	LN626295.1
		Penicillium javanicum strain WTS27 18S ribosomal RNA gene, partial sequence; internal transcribed spacer 1, 5.8S ribosomal RNA gene, and internal transcribed spacer 2, complete sequence; and 28S ribosomal RNA gene, partial sequence	972	972	100%	99.81%	GU966506.1
		Penicillium javanicum isolate Y9_ITS1F internal transcribed spacer 1, partial sequence; 5.8S ribosomal RNA gene and internal transcribed spacer 2, complete sequence; and large subunit ribosomal RNA gene, partial sequence	968	968	99%	99.81%	MF574327.1
		Penicillium javanicum strain CBS 129771 small subunit ribosomal RNA gene, partial sequence; internal transcribed spacer 1, 5.8S ribosomal RNA gene, and internal transcribed spacer 2, complete sequence; and large subunit ribosomal RNA gene, partial sequence	966	966	100%	99.62%	MH865649.1
		Penicillium brefeldianum culture-collection CBS:233.81 internal transcribed spacer 1, 5.8S ribosomal RNA gene, and internal transcribed spacer 2, complete sequence; and 28S ribosomal RNA gene, partial sequence	965	965	99%	99.62%	GU981615.1
		Penicillium reticulisporum NRRL 3447 ITS region; from TYPE material	961	961	100%	99.43%	NR_121231.1
		Eupenicillium sp. CR-2009e internal transcribed spacer 1, partial sequence; 5.8S ribosomal RNA gene and internal transcribed spacer 2, complete sequence; and 28S ribosomal RNA gene, partial sequence	961	961	100%	99.43%	GU166451.1
		Penicillium reticulisporum strain NRRL 3447 internal transcribed spacer 1, 5.8S ribosomal RNA gene, and internal transcribed spacer 2, complete sequence; and 28S ribosomal RNA gene, partial sequence	961	961	100%	99.43%	AF033437.1
**SBUG-M 1742 (17/P17/K1)**	*Penicillium javanicum*	*Penicillium javanicum* strain CBS 129395 small subunit ribosomal RNA gene, partial sequence; internal transcribed spacer 1, 5.8S ribosomal RNA gene, and internal transcribed spacer 2, complete sequence; and large subunit ribosomal RNA gene, partial sequence	957	957	100%	99.81%	MH865296.1

		Penicillium javanicum var. javanicum strain CBS 291.53 small subunit ribosomal RNA gene, partial sequence; internal transcribed spacer 1, 5.8S ribosomal RNA gene, and internal transcribed spacer 2, complete sequence; and large subunit ribosomal RNA gene, partial sequence	957	957	100%	99.81%	MH857207.1
		Penicillium javanicum strain WTS27 18S ribosomal RNA gene, partial sequence; internal transcribed spacer 1, 5.8S ribosomal RNA gene, and internal transcribed spacer 2, complete sequence; and 28S ribosomal RNA gene, partial sequence	957	957	100%	99.81%	GU966506.1
		Eupenicillium sp. K4 internal transcribed spacer 1, 5.8S ribosomal RNA gene, and internal transcribed spacer 2, complete sequence; and 28S ribosomal RNA gene, partial sequence	955	955	99%	99.81%	KJ191440.1
		Penicillium javanicum strain CBS 129771 small subunit ribosomal RNA gene, partial sequence; internal transcribed spacer 1, 5.8S ribosomal RNA gene, and internal transcribed spacer 2, complete sequence; and large subunit ribosomal RNA gene, partial sequence	952	952	100%	99.81%	MH865649.1
		Penicillium javanicum isolate Y9_ITS1F internal transcribed spacer 1, partial sequence; 5.8S ribosomal RNA gene and internal transcribed spacer 2, complete sequence; and large subunit ribosomal RNA gene, partial sequence	950	950	99&	99.62%	MF574327.1
		Penicillium reticulisporum NRRL 3447 ITS region; from TYPE material	946	946	100%	99.81%	NR_121231.1
		Eupenicillium sp. CR-2009f internal transcribed spacer 1, partial sequence; 5.8S ribosomal RNA gene and internal transcribed spacer 2, complete sequence; and 28S ribosomal RNA gene, partial sequence	946	946	100%	99.42%	GU166462.1
		Eupenicillium sp. CR-2009e internal transcribed spacer 1, partial sequence; 5.8S ribosomal RNA gene and internal transcribed spacer 2, complete sequence; and 28S ribosomal RNA gene, partial sequence	946	946	100%	99.42%	GU166451.1
		Penicillium reticulisporum strain NRRL 3447 internal transcribed spacer 1, 5.8S ribosomal RNA gene, and internal transcribed spacer 2, complete sequence; and 28S ribosomal RNA gene, partial sequence	946	946	100%	99.42%	AF033437.1
**SBUG-M 1747 (11/P27/K1)**	*Fusarium oxysporum*	*Fusarium oxysporum* f. sp. ciceris small subunit ribosomal RNA gene, partial sequence; internal transcribed	891	891	100%	100%	MK074845.1
		Fusarium fujikuroi var. moniliformis isolate Y-072 small subunit ribosomal RNA gene, partial sequence; internal transcribed spacer 1, 5.8S ribosomal RNA gene, and internal transcribed spacer 2, complete sequence; and large subunit ribosomal RNA gene, partial sequence	891	951	100%	100%	MN565957.1
		Fusarium culmorum isolate y-018 internal transcribed spacer 1, partial sequence; 5.8S ribosomal RNA gene and internal transcribed spacer 2, complete sequence; and large subunit ribosomal RNA gene, partial sequence	891	891	100%	100%	MN565954.1

		Fusarium oxysporum isolate y-005 internal transcribed spacer 1, partial sequence; 5.8S ribosomal RNA gene and internal transcribed spacer 2, complete sequence; and large subunit ribosomal RNA gene, partial sequence	891	891	100%	100%	MN560031.1
		Fusarium oxysporum isolate y-003 internal transcribed spacer 1, partial sequence; 5.8S ribosomal RNA gene and internal transcribed spacer 2, complete sequence; and large subunit ribosomal RNA gene, partial sequence	891	891	100%	100%	MN559984.1
		Fusarium oxysporum strain FO44 internal transcribed spacer 1, partial sequence; 5.8S ribosomal RNA gene and internal transcribed spacer 2, complete sequence; and large subunit ribosomal RNA gene, partial sequence	891	891	100%	100%	MN538904.1
		Fusarium oxysporum strain RSF-2018.5.16-#2 small subunit ribosomal RNA gene, partial sequence; internal transcribed spacer 1, 5.8S ribosomal RNA gene, and internal transcribed spacer 2, complete sequence; and large subunit ribosomal RNA gene, partial sequence	891	891	100%	100%	MK463988.1
		Fusarium oxysporum f. sp. cubense isolate Ollukkara - Thrissur TSR 5 small subunit ribosomal RNA gene, partial sequence; internal transcribed spacer 1, 5.8S ribosomal RNA gene, and internal transcribed spacer 2, complete sequence; and large subunit ribosomal RNA gene, partial sequence	891	891	100%	100%	MN528565.1
		Fusarium oxysporum f. sp. cubense isolate Chalakudy - Thrissur TSR 3 small subunit ribosomal RNA gene, partial sequence; internal transcribed spacer 1, 5.8S ribosomal RNA gene, and internal transcribed spacer 2, complete sequence; and large subunit ribosomal RNA gene, partial sequence	891	891	100%	100%	MN527522.1
		Fusarium oxysporum f. sp. cubense isolate Aluva - Ernakulam EKM 3 small subunit ribosomal RNA gene, partial sequence; internal transcribed spacer 1, 5.8S ribosomal RNA gene, and internal transcribed spacer 2, complete sequence; and large subunit ribosomal RNA gene, partial sequence	891	891	100%	100%	MN527256.1
**SBUG-M 1749 (68/P38/K1)**	*Scedosporium boydii*	*Scedosporium boydii* strain CNRMA5.1128 isolate ISHAM-ITS_ID MITS2336 18S ribosomal RNA gene, partial sequence; internal transcribed spacer 1, 5.8S ribosomal RNA gene, and internal transcribed spacer 2, complete sequence; and 28S ribosomal RNA gene, partial sequence	1020	1020	100%	100%	KP132690.1
		Pseudallescheria ellipsoidea isolate LMA 80677953/1 18S ribosomal RNA gene, partial sequence; internal transcribed 1, 5.8S ribosomal RNA, and internal transcribed 2 genes, complete sequence; and 28S ribosomal RNA gene, partial sequence	1020	1020	100%	100%	JQ690937.1
		Pseudallescheria ellipsoidea isolate LMA 40000584 18S ribosomal RNA gene, partial sequence; internal transcribed 1, 5.8S ribosomal RNA, and internal transcribed 2 genes, complete sequence; and 28S ribosomal RNA gene, partial sequence	1020	1020	100%	100%	JQ690930.1

		Pseudallescheria ellipsoidea isolate LMA 40407486 18S ribosomal RNA gene, partial sequence; internal transcribed 1, 5.8S ribosomal RNA, and internal transcribed 2 genes, complete sequence; and 28S ribosomal RNA gene, partial sequence	1020	1020	100%	100%	JQ690911.1
		Pseudallescheria boydii strain 04730 internal transcribed spacer 1, partial sequence; 5.8S ribosomal RNA gene and internal transcribed spacer 2, complete sequence; and 28S ribosomal RNA gene, partial sequence	1020	1020	100%	100%	JN207447.1
		Pseudallescheria boydii strain 01116 internal transcribed spacer 1, partial sequence; 5.8S ribosomal RNA gene and internal transcribed spacer 2, complete sequence; and 28S ribosomal RNA gene, partial sequence	1020	1020	100%	100%	JN207439.1
		Pseudallescheria boydii strain UWFP 823 18S ribosomal RNA gene, partial sequence; internal transcribed spacer 1, 5.8S ribosomal RNA gene, and internal transcribed spacer 2, complete sequence; and 28S ribosomal RNA gene, partial sequence	1020	1020	100%	100%	AY213683.1
		Pseudallescheria ellipsoidea isolate 42-3 18S ribosomal RNA gene, partial sequence; internal transcribed spacer 1, 5.8S ribosomal RNA gene, and internal transcribed spacer 2, complete sequence; and 28S ribosomal RNA gene, partial sequence	1013	1013	100%	99.82%	KT223521.1
		Scedosporium boydii strain SA9 isolate ISHAM-ITS_ID MITS2335 18S ribosomal RNA gene, partial sequence; internal transcribed spacer 1, 5.8S ribosomal RNA gene, and internal transcribed spacer 2, complete sequence; and 28S ribosomal RNA gene, partial sequence	1013	1013	99%	100%	KP132698.1
		Scedosporium boydii strain C. C. Lee AgFN4-8-2 small subunit ribosomal RNA gene, partial sequence; internal transcribed spacer 1, 5.8S ribosomal RNA gene, and internal transcribed spacer 2, complete sequence; and large subunit ribosomal RNA gene, partial sequence	1009	1009	100%	99.64%	MH793590.1
**SBUG-M 1751 (71/P36/K1)**	*Purpureocillium lilacinum*	*Purpureocillium* sp. strain Mal02-Mod small subunit ribosomal RNA gene, partial sequence; internal transcribed spacer 1, 5.8S ribosomal RNA gene, and internal transcribed spacer 2, complete sequence; and large subunit ribosomal RNA gene, partial sequence	1051	1051	100%	100%	MH426603.1
		Purpureocillium lilacinum 18S ribosomal RNA gene, partial sequence; internal transcribed spacer 1, 5.8S ribosomal RNA gene, and internal transcribed spacer 2, complete sequence; and 28S ribosomal RNA gene, partial sequence	1051	1051	100%	100%	KJ938575.1
		Purpureocillium lilacinum isolate F129 small subunit ribosomal RNA gene, partial sequence; internal transcribed spacer 1, 5.8S ribosomal RNA gene, and internal transcribed spacer 2, complete sequence; and large subunit ribosomal RNA gene, partial sequence	1040	1040	100%	99.65%	MN242828.1
		Purpureocillium lilacinum isolate CES3 small subunit ribosomal RNA gene, partial sequence;	1040	1040	100%	99.65%	MN173144.1

internal transcribed spacer 1, 5.8S ribosomal RNA gene, and internal transcribed spacer 2, complete sequence; and large subunit ribosomal RNA gene, partial sequence					
Purpureocillium lilacinum strain KUYZ001B-1-3 internal transcribed spacer 1, partial sequence; 5.8S ribosomal RNA gene and internal transcribed spacer 2, complete sequence; and large subunit ribosomal RNA gene, partial sequence	1040	1040	100%	99.65%	MH477737.1
Purpureocillium sp. isolate 30 small subunit ribosomal RNA gene, partial sequence; internal transcribed spacer 1, 5.8S ribosomal RNA gene, and internal transcribed spacer 2, complete sequence; and large subunit ribosomal RNA gene, partial sequence	1040	1040	100%	99.65%	KY318491.1
Purpureocillium sp. isolate SR50 small subunit ribosomal RNA gene, partial sequence; internal transcribed spacer 1, 5.8S ribosomal RNA gene, and internal transcribed spacer 2, complete sequence; and large subunit ribosomal RNA gene, partial sequence	1040	1040	100%	99.65%	KX009140.1
Purpureocillium sp. isolate SR20 internal transcribed spacer 1, partial sequence; 5.8S ribosomal RNA gene and internal transcribed spacer 2, complete sequence; and large subunit ribosomal RNA gene, partial sequence	1040	1040	100%	99.65%	KX009123.1
Penicillium sp. isolate SR15 internal transcribed spacer 1, partial sequence; 5.8S ribosomal RNA gene and internal transcribed spacer 2, complete sequence; and large subunit ribosomal RNA gene, partial sequence	1040	1040	100%	99.65%	KX009119.1
Purpureocillium sp. xz16 18S ribosomal RNA gene, partial sequence; internal transcribed spacer 1, 5.8S ribosomal RNA gene, and internal transcribed spacer 2, complete sequence; and 28S ribosomal RNA gene, partial sequence	1040	1040	100%	99.65%	KJ935014.1

a) first ten hits of the NCBI (National Centre for Biotechnology Information) BLAST nucleotide collection (ITS) are presented

Table S3. Results of identification of the isolated filamentous fungi by ITS gene sequence analysis in Mycobank ITS database.

**Isolate**	**Result of identification**	**ITS gene sequence analysis**[a)]							
		Reference description	Score	Probability	Similarity %	Fragments	Overlap %	Direction[b)]	Rating[c)]
**SBUG-M 1741 (15/P17/K2)**	*Penicillium javanicum*	CBS 291.53 *Eupenicillium javanicum* var. javanicum, *Eupenicillium javanicum* var. javanicum, Filamentous fungi, Germany, soil, publicly available rDNA ITS sequences (nlink4056)	835.28	0	99.81	1	100	+/+	****
		SH1529987.08FU LN626295 Fungi, Ascomycota, Eurotiomycetes, Eurotiales, Aspergillaceae, Penicillium, *Penicillium* sp	835.28	0	99.81	1	100	+/+	****
		SH1529987.08FU GU966506 Fungi, Ascomycota, Eurotiomycetes, Eurotiales, Aspergillaceae, Penicillium, *Penicillium javanicum*	835.28	0	99.81	1	100	+/+	****
		SH1529987.08FU MF574327 Fungi, Ascomycota, Eurotiomycetes, Eurotiales, Aspergillaceae, Penicillium, *Penicillium javanicum*	824.18	0	99.81	1	98.67	+/+	****
		CBS 283.36 CBS 283.36_ex31227_33058_ITS *Eupenicillium javanicum* var. javanicum, *Eupenicillium javanicum* var. javanicum, Filamentous fungi, Sumatra, soil, publicly available rDNA ITS sequences (nlink4056)	805.16	0	99.80	1	96.40	+/-	****
		MIRRI0042673 CBS 283.36_ex31227_33058_ITS CBS 283.36, soil, *Eupenicillium javanicum* var. javanicum, its: Sequences ITS	805.16	0	99.80	1	96.40	+/-	****
		SH1529987.08FU MF574328 Fungi, Ascomycota, Eurotiomycetes, Eurotiales, Aspergillaceae, Penicillium, *Penicillium javanicum*	795.65	0	99.80	1	95.27	+/+	****
		SH1529987.08FU FJ231024 Fungi, Ascomycota, Eurotiomycetes, Eurotiales, Aspergillaceae, Penicillium, *Penicillium malacosphaerulum*	822.60	0	99.62	1	98.86	+/+	****
		SH1529987.08FU FJ231026 Fungi, Ascomycota, Eurotiomycetes, Eurotiales, Aspergillaceae, Penicillium, *Penicillium malacosphaerulum*	822.60	0	99.62	1	98.86	+/+	****
		SH1529987.08FU GU981615 Fungi, Ascomycota, Eurotiomycetes, Eurotiales, Aspergillaceae, Penicillium, *Penicillium dodgei*	822.60	0	99.62	1	98.86	+/+	****

**SBUG-M 1742 (17/P17/K1)**	*Penicillium javanicum*	CBS 291.53 *Eupenicillium javanicum* var. javanicum, *Eupenicillium javanicum* var. javanicum, Filamentous fungi, Germany, soil, publicly available rDNA ITS sequences (nlink4056)	824.18	0	99.81	1	100	+/+	****
		SH1529987.08FU GU966506 Fungi, Ascomycota, Eurotiomycetes, Eurotiales, Aspergillaceae, Penicillium, *Penicillium javanicum*	824.18	0	99.81	1	100	+/+	****
		SH1529987.08FU KJ191440 Fungi, Ascomycota, Eurotiomycetes, Eurotiales, Aspergillaceae, Penicillium, *Penicillium* sp.	822.60	0	99.81	1	99.81	+/+	****
		SH1529987.08FU MF574327 Fungi, Ascomycota, Eurotiomycetes, Eurotiales, Aspergillaceae, Penicillium, *Penicillium javanicum*	817.84	0	99.81	1	99.23	+/+	****
		SH1529987.08FU LN626295 Fungi, Ascomycota, Eurotiomycetes, Eurotiales, Aspergillaceae, Penicillium, *Penicillium* sp	813.09	0	99.81	1	98.66	+/+	****
		CBS 283.36 CBS 283.36_ex31227_33058_ITS *Eupenicillium javanicum* var. javanicum, *Eupenicillium javanicum* var. javanicum, Filamentous fungi, Sumatra, soil, publicly available rDNA ITS sequences (nlink4056)	743.35	0	99.79	1	90.21	+/-	****
		MIRRI0042673 CBS 283.36_ex31227_33058_ITS CBS 283.36, soil, *Eupenicillium javanicum* var. javanicum, its: Sequences ITS	743.35	0	99.79	1	90.21	+/-	****
		SH1529987.08FU GU981615 Fungi, Ascomycota, Eurotiomycetes, Eurotiales, Aspergillaceae, Penicillium, *Penicillium dodgei*	809.92	0	99.61	1	98.66	+/+	****
		SH1529987.08FU GU981616 Fungi, Ascomycota, Eurotiomycetes, Eurotiales, Aspergillaceae, Penicillium, *Penicillium decumbens*	809.92	0	99.61	1	98.66	+/+	****
		CBS 233.81 CBS23381_2825_ITS *Eupenicillium brefeldianum, Eupenicillium brefeldianum,* Filamentous fungi, Neotype of *Penicillium brefeldianum* B.O. Dodge, Australia, publicly available rDNA ITS sequences (nlink4056)	759.20	0	99.59	1	92.51	+/+	****

**SBUG-M 1747 (11/P27/K1)**	*Fusarium oxyporum*	LC13769 MW016603 *Fusarium* sp. in FOSC (Wang et al. 2022), *F. oxysporum* species complex (FOSC), LC13769 = LJM1259-3, China, Passiflora edulis, ITS (n4)	765.54	0	100	1	100	+/+	****
		CBS 144742, CBS 144742 = CPC 25801 ITS *Fusarium fabacearum*, *F. oxysporum* species complex (FOSC), CBS 144742 = CPC 25801, South Africa, Zea mays, ITS (n4)	765.54	0	100	1	100	+/+	****
		CBS 110286, MH862858 *Fusarium* foetens, *F. oxysporum* species complex (FOSC), ex-type of *Fusarium foetens*, CBS 110286 = NRRL 31852 = PD 2001/7244, Netherlands, Begonia elatior hybrid, causing tracheomycosis, ITS (n4)	765.54	0	100	1	100	+/+	****
		CBS 130301 MH865885 *Fusarium nirenbergiae*, *F. oxysporum* species complex (FOSC), CBS 130301 = NRRL 26374, United States of America, Human leg ulcer, ITS (n4)	765.54	0	100	1	100	+/+	****
		BE19-004016 MZ890521 *Fusarium nirenbergiae*, *F. oxysporum* species complex (FOSC), BE19-004016, Belgium, Garden soil, ITS (n4)	765.54	0	100	1	100	+/+	****
		NL19-053002 MZ890529 *Fusarium nirenbergia*e, *F. oxysporum* species complex (FOSC), NL19-053002, Netherlands, Garden soil, ITS (n4)	765.54	0	100	1	100	+/+	****
		JW 288013, MZ890525 *Fusarium nirenbergiae*, *F. oxysporum* species complex (FOSC), JW 288013, Netherlands, Garden soil, ITS (n4)	765.54	0	100	1	100	+/+	****
		JW 288013 MZ890525 *Fusarium nirenbergiae*, *F. oxysporum* species complex (FOSC), JW 288013, Netherlands, Garden soil, ITS (n4)	765.54	0	100	1	100	+/+	****

		NL19-94002 MZ890538 *Fusarium oxysporum*, *F. oxysporum* species complex (FOSC), NL19-94002, Netherlands, Garden soil, ITS (n4)	765.54	0	100	1	100	+/+	****
		NL19-94008 MZ890539 *Fusarium oxysporum*, *F. oxysporum* species complex (FOSC), NL19-94008, Netherlands, Garden soil, ITS (n4)	765.54	0	100	1	100	+/+	****
**SBUG-M 1749 (68/P38/K1)**	*Scedosporium boydii*	CNRMA16.348 *Scedosporium boydii*, n3: rDNA sequences ITS	876.48	0	100	1	100	+/+	****
		CNRMA19.196 *Scedosporium ellipsoideum*, n3: rDNA sequences ITS	876.48	0	100	1	100	+/+	****
		CNRMA17.591 *Scedosporium ellipsoideum, n3: rDNA sequences ITS*	876.48	0	100	1	100	+/+	****
		CNRMA17.591 *Scedosporium ellipsoideum*, n1: rDNA sequences 28S	876.48	0	100	1	100	+/+	****
		CNRMA16.529 *Scedosporium ellipsoideum*, n3: rDNA sequences ITS	876.48	0	100	1	100	+/+	****
		CNRMA16.178 *Scedosporium ellipsoideum*, n3: rDNA sequences ITS	876.48	0	100	1	100	+/+	****
		CNRMA5.1128 CNRMA5.1128 - rDNA sequences ITS - Id#41036 *Scedosporium ellipsoideum*, n3: rDNA sequences ITS	876.48	0	100	1	100	+/+	****
		CBS 127623 CBS_127623-13861_ITS4_D8_058 *Scedosporium apiospermum*, *Scedosporium apiospermum*, Filamentous fungi, China, man;Cerebrospinal fluid, publicly available rDNA ITS sequences (nlink4056)	816.26	0	100	1	93.12	+/-	****
		CBS 418.73 *Pseudallescheria ellipsoidea*, *Pseudallescheria ellipsoidea*, Filamentous fungi, Type of *Petriellidium ellipsoideum* Arx & Fassat., Tajikistan, soil, publicly available rDNA ITS sequences (nlink4056)	816.26	0	100	1	93.12	+/+	****
		MIRRI0080469 CBS_127623-13861_ITS4_D8_058 CBS 127623, man;Cerebrospinal fluid, China, *Scedosporium apiospermum*, its: Sequences ITS	816.26	0	100	1	93.12	+/-	****

**SBUG-M 1751 (71/P36/K1)**	*Purpureocillium lilacinum*	CNRMA18.195 *Purpureocillium lilacinum*, n3: rDNA sequences ITS	901.84	0	100	1	99.82	+/+	****
		SH1561003.08FU MH426603 Fungi, Ascomycota, Sordariomycetes, Hypocreales, Ophiocordycipitaceae, Purpureocillium, *Purpureocillium* sp	903.43	0	100	1	100	+/+	****
		SH1561003.08FU KR909221 Fungi, Ascomycota, Sordariomycetes, Hypocreales, Ophiocordycipitaceae, Purpureocillium, *Purpureocillium lilacinum*	903.43	0	100	1	100	+/+	****
		SH1561003.08FU KJ938575 Fungi, Ascomycota, Sordariomycetes, Hypocreales, Ophiocordycipitaceae, Purpureocillium, *Purpureocillium lilacinum*	903.43	0	100	1	100	+/+	****
		SH1561003.08FU AY213667 Fungi, Ascomycota, Sordariomycetes, Hypocreales, Ophiocordycipitaceae, Purpureocillium, *Purpureocillium lilacinum*	897.09	0	99.65	1	100	+/+	****
		SH1561003.08FU AB244777 Fungi, Ascomycota, Sordariomycetes, Hypocreales, Ophiocordycipitaceae, Purpureocillium, *Purpureocillium lilacinum*	897.09	0	99.65	1	100	+/+	****
		SH1561003.08FU KY318491 Fungi, Ascomycota, Sordariomycetes, Hypocreales, Ophiocordycipitaceae, Purpureocillium, *Purpureocillium* sp	897.09	0	99.65	1	100	+/+	****
		SH1561003.08FU KJ935014 Fungi, Ascomycota, Sordariomycetes, Hypocreales, Ophiocordycipitaceae, Purpureocillium, *Purpureocillium* sp	897.09	0	99.65	1	100	+/+	****
		SH1561003.08FU KX009123 Fungi, Ascomycota, Sordariomycetes, Hypocreales, Ophiocordycipitaceae, Purpureocillium, *Purpureocillium* sp	897.09	0	99.65	1	100	+/+	****
		SH1561003.08FU KX009119 Fungi, Ascomycota, Sordariomycetes, Hypocreales, Ophiocordycipitaceae, Purpureocillium, *Purpureocillium* sp	897.09	0	99.65	1	100	+/+	****

a) first ten hits of the mycobank analysis are presented.
b) +/+, presents similar direction of the query (submitted) and database sequences; while +/- indicates different direction of the submitted sequence from the database hit (reverse-complement counterpart). .
c) mycobank ITS database presents the overall quality of the blast result in a range from bad (*) to excellent (*****). Here only good (****) rating is detected for all hits.

Table S4. Results of identification of the isolated filamentous fungi by 18S gene sequence analysis in the NCBI SSU database.

Isolate	Result of identification	18S gene sequence analysis[a)]					
		Description	Max score	Total score	Query cover	Ident.	Accession
**SBUG-M 1741 (15/P17/K2)**	*Penicillium* sp. *(javanicum)*	*Penicillium decumbens* isolate XXTF9 small subunit ribosomal RNA gene, partial sequence	377	377	100%	100%	MN602644.1
		Penicillium chrysogenum strain MG2 small subunit ribosomal RNA gene, partial sequence	377	377	100%	100%	MN453365.1
		Penicillium sp. isolate FBP22 small subunit ribosomal RNA gene, partial sequence	377	377	100%	100%	MN396379.1
		Penicillium sp. isolate FBP3 small subunit ribosomal RNA gene, partial sequence	377	377	100%	100%	MN396378.1
		Penicillium sp. isolate FBP1 small subunit ribosomal RNA gene, partial sequence	377	377	100%	100%	MN396377.1
		Penicillium sp. strain M30 18S ribosomal RNA gene, partial sequence	377	377	100%	100%	MH673731.1
		Penicillium sp. strain M26 18S ribosomal RNA gene, partial sequence	377	377	100%	100%	MH673730.1
		Penicillium sp. MA-2019a isolate ShG4B small subunit ribosomal RNA gene, partial sequence	377	377	100%	100%	MK625187.1
		Penicillium malachiteum CBS 647.95 18S rRNA gene, partial sequence; from TYPE material	377	377	100%	100%	NG_062770.1
		Penicillium limosum CBS 339.97 18S rRNA gene, partial sequence; from TYPE material	377	377	100%	100%	NG_062729.1
**SBUG-M 1742 (17/P17/K1)**	*Penicillium* sp. *(javanicum)*	*Penicillium decumbens* isolate XXTF9 small subunit ribosomal RNA gene, partial sequence	364	364	100%	100%	MN602644.1
		Penicillium chrysogenum strain MG2 small subunit ribosomal RNA gene, partial sequence	364	364	100%	100%	MN453365.1
		Penicillium sp. isolate FBP22 small subunit ribosomal RNA gene, partial sequence	364	364	100%	100%	MN396379.1
		Penicillium sp. isolate FBP3 small subunit ribosomal RNA gene, partial sequence	364	364	100%	100%	MN396378.1
		Penicillium sp. isolate FBP1 small subunit ribosomal RNA gene, partial sequence	364	364	100%	100%	MN396377.1
		Penicillium sp. MA-2019a isolate ShG 4C small subunit ribosomal RNA gene, partial sequence	364	364	100%	100%	MK685358.1
		Penicillium sp. strain M30 18S ribosomal RNA gene, partial sequence	364	364	100%	100%	MH673731.1
		Penicillium sp. strain M26 18S ribosomal RNA gene, partial sequence	364	364	100%	100%	MH673730.1
		Penicillium sp. MA-2019a isolate ShG4B small subunit ribosomal RNA gene, partial sequence	364	364	100%	100%	MK625187.1
		Penicillium oxalicum strain TGQM01 small subunit ribosomal RNA gene, partial sequence	364	364	100%	100%	MK069498.1

**SBUG-M 1747 (11/P27/K1)**	*Fusarium oxysporum*	*Fusarium solani* isolate XXTF8 small subunit ribosomal RNA gene, partial sequence	375	375	100%	100%	MN602643.1
		Fusarium sp. MAS2 strain SZBNS18 small subunit ribosomal RNA gene, partial sequence	375	375	100%	100%	MN508437.1
		Fusarium sp. isolate RT18 small subunit ribosomal RNA gene, partial sequence	375	375	100%	100%	MN461254.1
		Fungal sp. isolate RT17 small subunit ribosomal RNA gene, partial sequence	375	375	100%	100%	MN461253.1
		Fusarium sp. isolate RT15 small subunit ribosomal RNA gene, partial sequence	375	375	100%	100%	MN461252.1
		Fusarium oxysporum isolate UAM 1 small subunit ribosomal RNA gene, partial sequence	375	375	100%	100%	MN396369.1
		Fusarium sp. isolate F116 small subunit ribosomal RNA gene, partial sequence	375	375	100%	100%	MN240475.1
		Fusarium sp. isolate AHMF4 small subunit ribosomal RNA gene, partial sequence	375	375	100%	100%	MN094111.1
		Fusarium sp. isolate BPF6 small subunit ribosomal RNA gene, partial sequence	375	375	100%	100%	MK850377.1
		Fusarium proliferatum strain 144 small subunit ribosomal RNA gene, partial sequence; internal transcribed spacer 1, 5.8S ribosomal RNA gene, and internal transcribed spacer 2, complete sequence; and large subunit ribosomal RNA gene, partial sequence	375	375	100%	100%	MK828121.1
**SBUG-M 1749 (68/P38/K1)**	*Scedosporium boydii Synonym Pseudallescheria boydii*	*Pseudallescheria ellipsoidea* CBS 418.73 18S rRNA gene, partial sequence; from TYPE material	379	379	100%	100%	NG_063099.1
		Pseudallescheria sp. MJ-X15 18S ribosomal RNA gene, partial sequence	379	379	100%	100%	HM590664.1
		Pseudallescheria sp. x13-118 18S ribosomal RNA gene, partial sequence	379	379	100%	100%	HQ234234.1
		Pseudallescheria sp. T55 partial 18S rRNA gene, strain T55	379	379	100%	100%	FN666094.1
		Pseudallescheria sp. D42 18S ribosomal RNA gene, partial sequence	379	379	100%	100%	GU183392.1
		Pseudallescheria ellipsoidea 18S ribosomal RNA gene, partial sequence	379	379	100%	100%	U43911.1
		Pseudallescheria boydii 18S ribosomal RNA gene, partial sequence	379	379	100%	100%	U43914.1
		Pseudallescheria boydii 18S ribosomal RNA gene, partial sequence	379	379	100%	100%	U43913.1
		Pseudallescheria boydii 18S ribosomal RNA gene, partial sequence	379	379	100%	100%	U43912.1
		Pseudalllescheria boydii partial 18S ribosomal RNA sequence	379	379	100%	100%	M89782.1
**SBUG-M 1751 (71/P36/K1)**	*Purpureocillium lilacinum Synomym Paecilomyces lilacinus*	*Purpureocillium lilacinum* strain PLBJ-1 18S ribosomal RNA gene, partial sequence	377	377	99%	100%	KM453730.1
		Purpureocillium lilacinum strain 5H1-S3-P4-3 18S ribosomal RNA gene, partial sequence	377	377	99%	100%	KM222279.1
		Purpureocillium sp. 4H1_P0_P1_2 strain 4H1-P0-P1-2 18S ribosomal RNA gene, partial sequence	377	377	99%	100%	KM222253.1

	Purpureocillium sp. 1H3_M3_P1_2 strain 1H3-M3-P1-2 18S ribosomal RNA gene, partial sequence	377	377	99%	100%	KM222228.1
	Paecilomyces lilacinus strain TS02 18S ribosomal RNA gene, partial sequence	377	377	99%	100%	FJ941875.1
	Paecilomyces lilacinus strain PF05 18S ribosomal RNA gene, partial sequence	377	377	99%	100%	FJ941854.1
	Paecilomyces hepiali strain Ph-4Qinghai 18S ribosomal RNA gene, partial sequence; internal transcribed spacer 1, 5.8S ribosomal RNA gene, and internal transcribed spacer 2, complete sequence; and 28S ribosomal RNA gene, partial sequence	377	377	99%	100%	EF555097.3
	Paecilomyces lilacinus strain M1 18S ribosomal RNA gene, partial sequence	377	377	99%	100%	FJ461772.1

a) first hits of the corresponding genus of the NCBI (National Centre for Biotechnology Information) BLAST nucleotide collection (SSU) are presented.

Table S5. Growth experiment of SBUG-M 1741, SBUG-M1742 and SBUG-M 1749 on tetradecane after 5 and 7 days.

Strain	Method		Growth time 5d	Growth time 7d		Growth time 5d	Growth time 7d
P.javanicum SBUG-M 1741	Three-point	First biological replicate	+++	+++	Second biological replicate	+++	+++
	Isolation streak		+++	+++		+++	+++
	Control		+	+		+	+
P.javanicum SBUG-M 1742	Three-point		+++	+++		+++	+++
	Isolation streak		++	+++		+++	+++
	Control		+	+		+	+
S.boydii SBUG-M 1749	Three-point		++	++		++	++
	Isolation streak		++	++		+	+
	Control		++	++		++	++

+ weak growth; ++ good growth, +++ strong growth

Table S6. Growth experiments of SBUG-M 1747 and SBUG-M 1751 on pristane after 5 and 7 days*.

Strain	Method		Growth time			Growth time	
			5d	7d		5d	7d
F.oxysporum SBUG-M 1747	Three-point	First biological replicate	+	+	Second biological replicate	+	+
	Isolation streak		+	+		+	+
	Control		+	+		+	+
P. lilacinum SBUG-M 1751	Three-point		+	+		+	+
	Isolation streak		+	+		+	+
	Control		+	+		+	+

+ weak growth
*Extending the experiment until 20 days showed the same results.
